# Mangiferin Prevents TBHP-Induced Apoptosis and ECM Degradation in Mouse Osteoarthritic Chondrocytes via Restoring Autophagy and Ameliorates Murine Osteoarthritis

**DOI:** 10.1155/2019/8783197

**Published:** 2019-10-15

**Authors:** Yao Li, Yaosen Wu, Kaixia Jiang, Wen Han, Jing Zhang, Lin Xie, Yanlong Liu, Jian Xiao, Xiangyang Wang

**Affiliations:** ^1^Department of Orthopaedics, The Second Affiliated Hospital and Yuying Children's Hospital of Wenzhou Medical University, Wenzhou, Zhejiang, China; ^2^Molecular Pharmacology Research Center, School of Pharmaceutical Science, Wenzhou Medical University, Wenzhou, Zhejiang, China; ^3^The Second School of Medicine, Wenzhou Medical University, Wenzhou, Zhejiang, China

## Abstract

Osteoarthritis (OA) is an age-related degenerative disease with complicated pathology involving chondrocyte apoptosis and extracellular matrix (ECM) degradation. Previous studies have shown that moderate autophagy has a protective effect against apoptosis in chondrocyte. Mangiferin is a natural polyphenol and exerts multiple pharmacological effects on different diseases in various preclinical studies. In this study, we investigated the effects of mangiferin on OA and delineated a potential molecular mechanism. In vitro, mangiferin treatment inhibited the expression of proapoptotic proteins induced by *tert*-butyl hydroperoxide (TBHP), increased the expression of antiapoptotic Bcl-2, and prevented ECM degradation by inhibiting the production of matrix-degrading enzyme. Mechanistically, mangiferin enhanced autophagy by activating the AMP-activated protein kinase (AMPK) signaling pathway. On the contrary, inhibition of autophagy partly abolished the protective effects of mangiferin on antiapoptosis and ECM synthesis in TBHP-treated chondrocyte. Correspondingly, the protective effect of mangiferin was also found in a mouse OA model. In conclusion, our results suggested that mangiferin serves as a potentially applicable candidate for treating OA.

## 1. Introduction

Osteoarthritis (OA) is the most common form of degenerative joint disease accompanied by joint pain and dysfunction in the affected patients, which causes disability in the elderly and huge socioeconomic burden [1, 2]. Multiple pathological factors have been suggested to contribute to the onset and progression of OA, such as inflammatory response, reactive oxygen species, and cellular apoptosis, which result in cell senescence, loss of cell density, abnormal secretory activity, extracellular matrix (ECM) degradation, and aggravation of OA development in joint cartilage [3–6]. Meanwhile, as the only resident cells in the articular cartilage, chondrocyte plays a crucial role in the synthesis and turnover of ECM, which has been regarded as the essential components for maintaining the function and structure of cartilage [7]. Thus, maintaining chondrocyte in a healthy condition may be an available strategy for preventing the progression of cartilage degeneration.

Autophagy is a catabolic lysosomal response to various types of stress, in which cytoplasm structures, organelles, and macromolecules are digested and recycled to maintain cellular metabolism [8, 9]. Under hypoxia, nutrient deprivation, or other pathologic conditions, autophagy is activated to sustain energy and protein integrity and improve cellular survival and function through degrading dysfunctional intracellular components. In the development of OA, autophagy not only involves cartilage tissue morphogenesis in the joint but also has an important role in the chondrocyte and ECM homeostasis [10–13]. As the previous study reported, mice that were orthotopically intra-articularly injected with rapamycin showed improvement in cartilage degeneration in destabilized medial meniscus (DMM) [14], while mice deficient in ATG5 showed aggravated chondrocyte apoptosis and degeneration of cartilage in joints [15]. These reports mentioned that the activation of the autophagy of chondrocyte can protect the cell from stresses and promote chondrocyte survival in OA.

Mangiferin, a natural polyphenol with a C-glycosylxanthone structure, is mainly extracted from Mangifera indica, Anemarrhena asphodeloides, and Mangifera persiciformis and exerts cell protective effect under pathologic conditions, including anti-inflammatory and antioxidative [16–18]. Mangiferin is also proved to be associated with neuroprotection for the treatment of diabetic encephalopathy; the potential mechanisms of protection are related to the upregulating of glyoxalase-1 through Nrf2/ARE signaling [19]. Recently, a study has indicated that mangiferin enhances endochondral ossification-based bone repair through maintaining bone marrow-derived mesenchymal stem cell- (BMSC-) derived hypertrophic chondrocyte survival and sufficient hypertrophic differentiation and demonstrated the molecular mechanism of mangiferin is based on the activation of autophagy [20]. Although these previous studies have extensively investigated the function of mangiferin and supported this natural product as a promising drug, the effect of mangiferin on chondrocyte autophagy and its potential therapeutic function in OA patients remain unknown.

In this study, we investigated the effects of mangiferin on autophagy in chondrocyte under TBHP treatment and explored the underlying molecular mechanism. In addition, the potential therapeutic function of mangiferin was also evaluated in a DMM mouse model of OA.

## 2. Materials and Methods

### 2.1. Ethical Statement

All surgical interventions, treatments, and postoperative animal care procedures were conducted in strict accordance with the Animal Care and Use Committee of Wenzhou Medical University. No clinical trial was involved in the current study.

### 2.2. Reagents and Antibodies

Mangiferin (purity > 98%) was purchased from Beijing Solarbio Science & Technology (Beijing, China). *t*ert-Butyl hydroperoxide solution (TBHP), 3-methyladenine (3-MA), chloroquine (CQ), and type II collagenases were purchased from Sigma-Aldrich (St. Louis, MO, USA). Dorsomorphin (compound C) and bafilomycin A1 were purchased from MCE (Monmouth Junction, NJ, USA). The primary antibodies against Bcl-2, Bax, collagen II, MMP13, aggrecan, p62, p-AMPK, and lamp2 were obtained from Abcam (Cambridge, MA, USA); antibodies against AMPK, p-mTOR, and mTOR were purchased from Cell Signaling Technology (Danvers, MA, USA); antibody against ATG5 and GAPDH was acquired from Proteintech (Chicago, IL, USA); antibody against cleaved caspase3 was purchased from Affinity (Cincinnati, OH, USA); antibody against LC3 was purchased from Novus (Littleton, Co, USA). Alexa Fluor 488-labeled and Alexa Fluor 647-labeled goat anti-rabbit/mouse/rat secondary antibodies were purchased from Abcam (Cambridge, MA, USA). 4,6-Diamidino-2-phenylindole (DAPI) was obtained from Beyotime (Shanghai, China). The reagents for chondrocyte culture were obtained from Gibco (Grand Island, NY, USA).

### 2.3. Primary Mouse Chondrocyte Culture

Primary chondrocytes were isolated from eight C57BL/6 mice (4 males and 4 females, 2 weeks) by dissection of knee cartilages. Then, cartilages were entirely cut into pieces and incubated with 2 mg/mL of collagenase II in DMEM/F12 medium at 37°C for 4 h. After washing and suspension, chondrocytes were seeded on six-well plates in DMEM/F12 supplemented with 10% foetal bovine serum (FBS), 100 U/mL penicillin, and 100 *μ*g/mL streptomycin in 5% CO_2_ at 37°C. After the first 24 hours of incubation, culture media were replaced with fresh medium. When up to 80% confluency is achieved, chondrocytes were harvested by 0.25% Trypsin-EDTA and replanted into six-well culture plates at the appropriate density. The second passage cells were employed for all of our experiments and replaced with fresh media every 2 days thereafter until the experiments are finished.

### 2.4. Cell Viability Assay

The cytotoxicity of mangiferin on chondrocytes was evaluated by a cell counting kit-8 (CCK-8) assay (Dojindo Co., Kumamoto, Japan). According to the manufacturer's protocol, chondrocytes were seeded on 96-well plates (5^∗^10^4^ cells per well) and treated with mangiferin according to the experimental design. After reagent treatment, cells were washed with PBS, and then 10 *μ*L tetrazolium substrate was added in 100 *μ*L medium of each well and cultured at 37°C for 2 hours. The absorbance was measured at 450 nm using a microplate reader.

### 2.5. Animal Model

Sixty 10-week-old C57BL/6 male mice were purchased from the Animal Center of the Chinese Academy of Sciences (Shanghai, China) and housed under strict controlled environmental conditions. The osteoarthritis model was induced by surgical destabilization of the medial meniscus (DMM) as previously described [21]. Arthrotomy without the transaction of the medial meniscotibial ligament was performed in the left knee joint of mice, and these mice were used as the control group. After surgery, the mice were randomly divided into the following groups: sham group, DMM group, DMM+mangiferin treatment group, and autophagy inhibition group. After that, mice received mangiferin dissolved in CMC which was intragastrically administrated once a day (10 mg/kg) for eight consecutive weeks. Meanwhile, mice were injected with 3-MA (30 mg/kg) once a day for inhibition of autophagy [22, 23]. Mice in the vehicle groups were administered an equivalent volume of CMC. All animals were sacrificed after eight weeks postsurgery.

### 2.6. Western Blot Analysis

Proteins were isolated from chondrocytes by RIPA lysis buffer with 1 mM phenylmethanesulfonyl fluoride followed by 15 min centrifugation at 12000 r.p.m. at 4°C then were quantified by BCA reagents (Thermo, Rockford, IL, USA); equivalent amounts of proteins were separated by 8%-12% SDS-PAGE gels and transferred to polyvinylidene fluoride (PVDF) membranes (Bio-Rad, Hercules, CA, USA). PVDF membranes were blocked with 5% (*w*/*v*) nonfat milk in TBST (Tris-buffered saline with 0.1% Tween-20) for 120 minutes at room temperature and then incubated overnight at 4°C with the primary antibodies. Next, the PVDF membranes were washed with TBST and incubated with the secondary antibodies for 90 minutes at room temperature. All results were visualized by the ChemiDoc XRS+ Imaging System (Bio-Rad), and the bands were quantified using densitometric measurement by the Quantity One software.

### 2.7. Tissue Preparation and Histopathologic Analysis

The mice were killed by intraperitoneal injection of 10% chloral hydrate, and the knee joints were collected 8 weeks after surgery. Then, joints were fixed in 4% (*v*/*v*) paraformaldehyde for 24 h and decalcified in 10% (*v*/*v*) EDTA for 4 weeks. The tissues were dehydrated, embedded in paraffin, and cut into 5 *μ*m sagittal sections. Slides of each joint were stained with safranin O-fast green (S-O) and hematoxylin and eosin (H&E). The cellularity and morphology of cartilage and subchondral bone were examined by another group of experienced histology researchers in a blinded manner using a microscope and evaluated by using an Osteoarthritis Research Society International (OARSI) scoring system for medial femoral condyle and medial tibial plateaus described previously [24]. The severity of synovitis was graded using a scoring system as previously described [25]. Fifteen mice each group were used for histomorphometric scoring.

### 2.8. Immunofluorescence

Sagittal cartilage sections (0.5 *μ*m thickness) were deparaffinized, rehydrated, and washed three times for 15 minutes in PBS. Chondrocytes were washed in PBS, fixed in 4% paraformaldehyde, and permeated in 0.1% Triton X-100 for 15 minutes. Next, cells were blocked with 5% bovine serum albumin for 1 h at 37°C and rinsed with PBS and incubated with primary antibodies in a humid chamber overnight at 4°C. The chondrocytes were washed and incubated with Alexa Fluor 488- or Alexa Fluor 647-conjugated secondary antibodies for 1 h at 37°C and labeled with DAPI for 5 min. The images were observed by a Nikon ECLIPSE Ti microscope (Nikon, Tokyo, Japan).

### 2.9. TUNEL Staining

The level of DNA damage was detected by TUNEL staining in mouse cartilage. Then, cartilage sections were deparaffinized, rehydrated and incubated with 0.1% Triton X-100 for 30 minutes, and stained with the In Situ Cell Death Detection Kit (Yeasen Biochemical, Shanghai, China) according to the manufacturer's instructions for 30 min at 37°C, and the nuclei were stained with DAPI. Twenty-five fields of each slide were randomly selected and captured by a Nikon ECLIPSE Ti microscope (Nikon, Tokyo, Japan).

### 2.10. Statistical Analysis

All the data are presented as the mean ± standard deviation from at least three independent experiments. Statistical analyses were performed using SPSS statistical software program 19.0 (IBM, Armonk, NY, USA). Statistical analyses were performed using one-way analysis of variance (ANOVA), followed by Tukey's test for pairwise comparison. Nonparametric data (OARSI scores and synovitis scores) were analyzed by the Kruskal–Wallis *H* test. A *P* value < 0.05 was considered being significant.

## 3. Results

### 3.1. Effect of Mangiferin on Chondrocyte Viability

**Figure 1:**
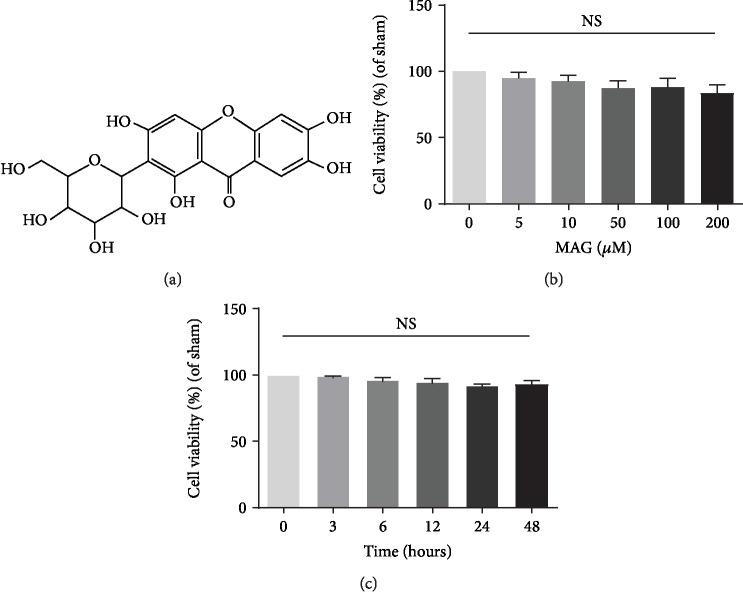


To evaluate the toxicity of mangiferin (Figure 1(a)) on chondrocytes, the CCK-8 assay was performed to detect the cell viability. Firstly, the cytotoxic effects of different concentrations of mangiferin (0, 5, 10, 50, 100, and 200 *μ*M) on chondrocytes were determined. Result indicated that mangiferin treatment presents no significant cytotoxicity at concentrations up to 200 *μ*m at 24 h (Figure 1(b)). Meanwhile, the cytotoxic effects of different time points (0, 3, 6, 12, 24, and 48 hours) of mangiferin at concentration with 100 *μ*m were also examined and showed no significant cytotoxicity from beginning to end hours (Figure 1(c)).

### 3.2. Effect of Mangiferin on Apoptosis in TBHP-Treated Chondrocytes

**Figure 2:**
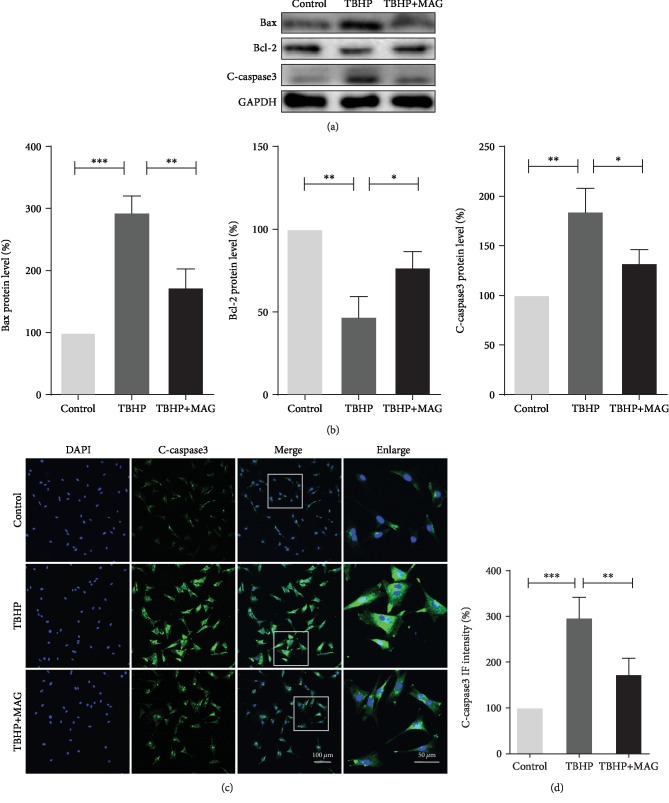


To determine the effect of mangiferin on apoptosis in mouse chondrocytes under TBHP-induced oxidative stress stimulation, the expression of Bax, Bcl-2, and cleaved caspase3 was detected by western blot and immunofluorescence staining. As shown in Figures 2(a) and 2(b), TBHP treatment upregulated the Bax and cleaved caspase3 expression and downregulated Bcl-2 expression. On the contrary, mangiferin significantly inhibited Bax and cleaved caspase3 expression and improved Bcl-2 expression. Similarly, the result of immunofluorescence staining labeled with cleaved caspase3 further confirmed that mangiferin treatment decreases the intensity of cleaved caspase3 (Figures 2(c) and 2(d)). These data indicate that mangiferin exerts antiapoptosis property in mouse chondrocytes.

### 3.3. Mangiferin Alleviates TBHP-Induced ECM Degradation in Chondrocytes

**Figure 3:**
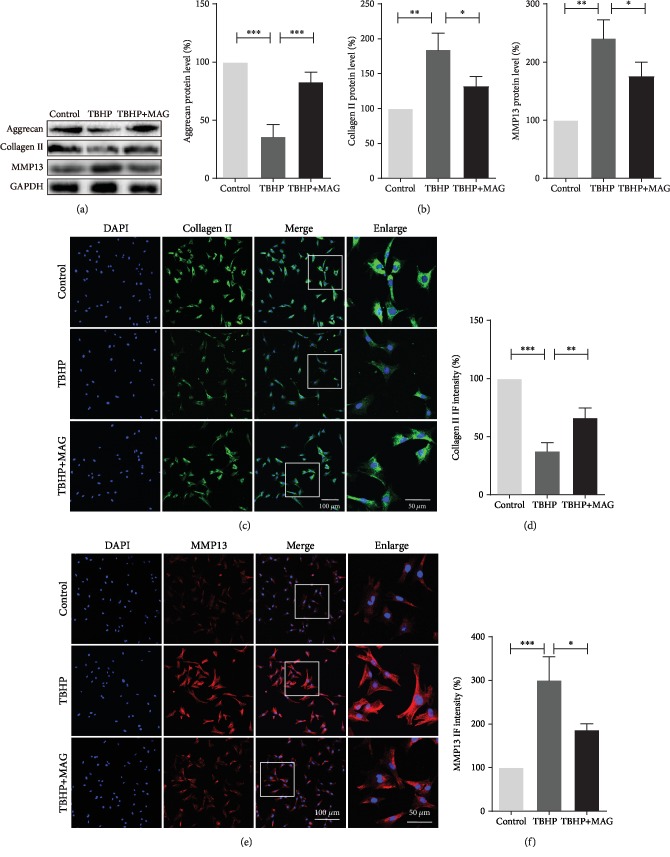


To evaluate the mangiferin function of TBHP-induced ECM degradation, we examined the collagen II, aggrecan, and MMP13 expression by western blot. As shown in Figures 3(a) and 3(b), TBHP treatment remarkably reduced aggrecan and collagen II synthesis but increased MMP13 expression. Instead, mangiferin pretreatment reversed the above destructive effects induced by TBHP. In addition, results of immunofluorescence staining labeled with collagen and MMP13 agreed with the western blot analysis as expected (Figures 3(c)–3(f)). Collectively, these results indicated that mangiferin possesses a protective effect on preventing ECM degradation.

### 3.4. Mangiferin Activates Autophagy and Enhances Autophagic Flux in Chondrocytes

**Figure 4:**
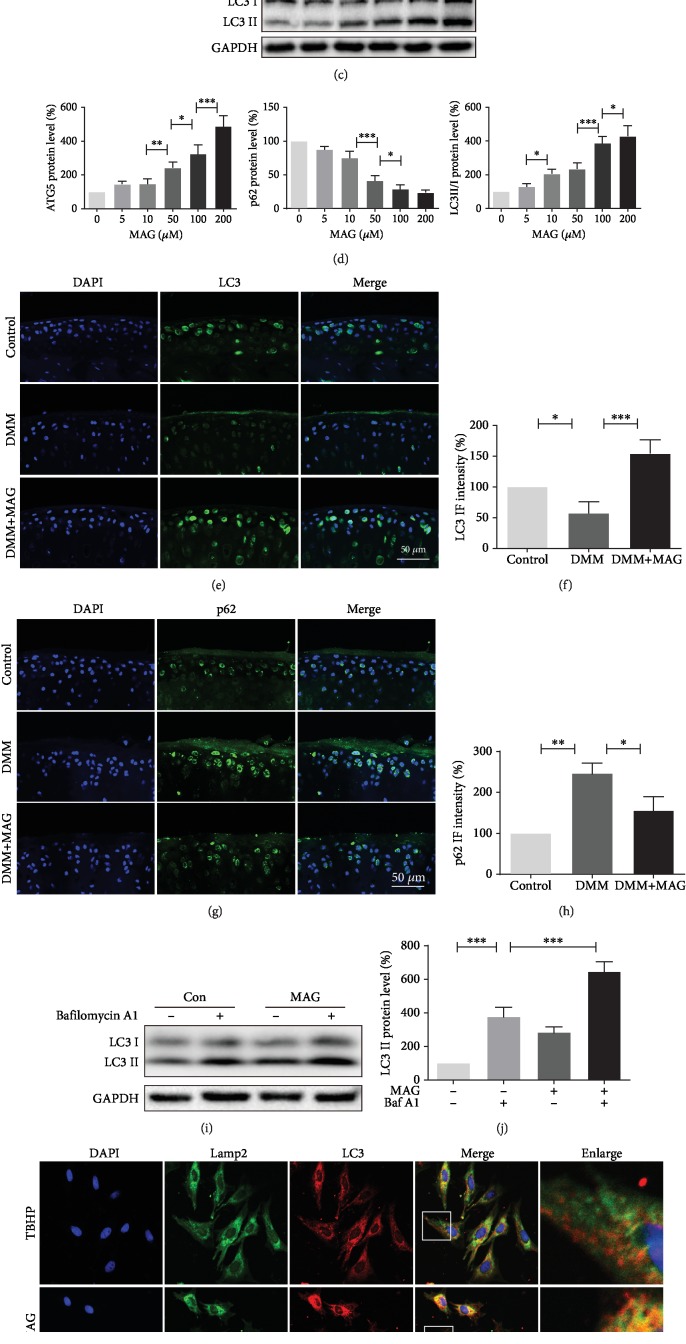


Autophagy is a common mechanism for the removal of excess or damaged organelles in the process of development and aging. Meanwhile, autophagy has a critical role in regulating energy recycle and cellular homoeostasis in chondrocytes. To investigate the effect whether mangiferin could activate autophagy in chondrocytes, autophagic markers, such as ATG5, p62, and LC3II/LC3I ratio, were examined in a dose- and time-dependent manner. Western blot analysis in a time-dependent manner presented that mangiferin treatment increases the LC3II/LC3I ratio and ATG5 accumulation and peaks at 24 hours. Contrarily, p62 expression was gradually decreased after treatment of mangiferin (Figures 4(a) and 4(b)). Besides, western blot analysis showed that mangiferin treatment results in increased ATG5 expression and LC3II/LC3I ratio but causes decreased p62 expression in a dose-dependent manner (Figures 4(c) and 4(d)). In addition, results of immunofluorescence staining showed that mangiferin treatment significantly increases the expression of LC3 (Figures 4(e) and 4(f)) and decreases the expression of p62 (Figures 4(g) and 4(h)) in the DMM model.

To detect whether mangiferin also enhances the state of autophagic flux, chondrocytes were treated with mangiferin and bafilomycin A1 [26]. Our results in Figures 4(i) and 4(j) showed the autophagic flux increased after mangiferin treatment. Furthermore, chondrocytes were colabeled with immunofluorescence staining for LC3 (a marker of autophagosome) and lamp2 (a marker of lysosome) after being treated with TBHP and TBHP and mangiferin, respectively. The intensity of LC3 and the fusion of autophagosome and lysosome in the TBHP and mangiferin treatment group were higher than those in the TBHP treatment group (Figures 4(k) and 4(l)). Therefore, all the above results indicate that mangiferin has a positive role during autophagy in chondrocyte.

### 3.5. Autophagy Enhanced by Mangiferin Depends on the Activating Level of p-AMPK

**Figure 5:**
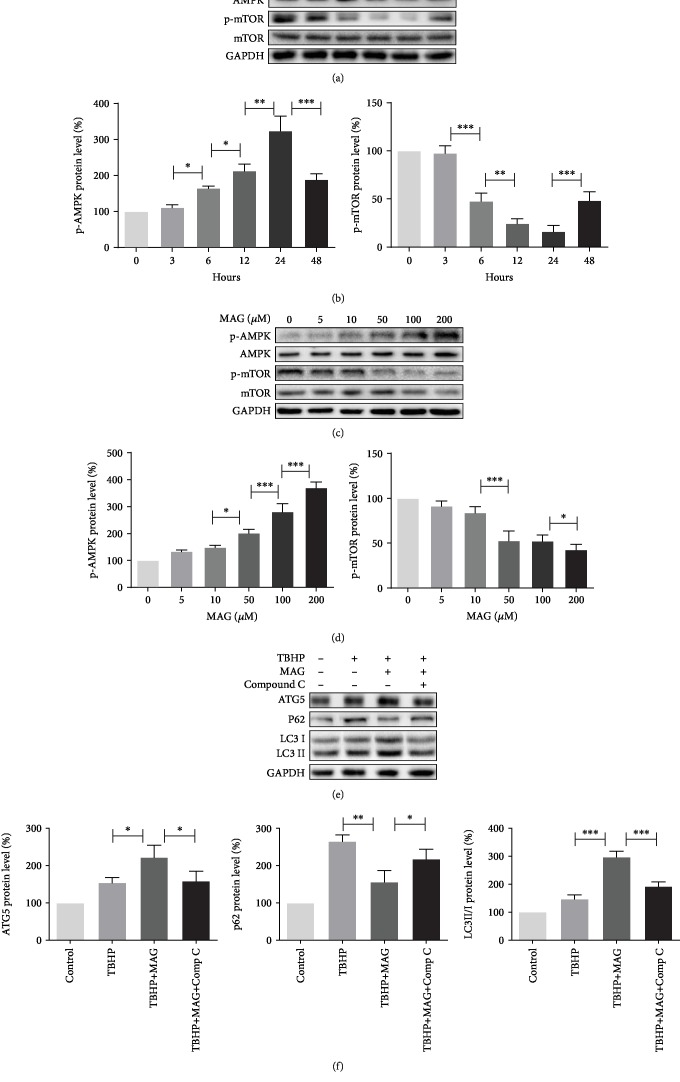


It is well accepted that the AMPK signal pathway is a critical regulator for autophagy, and downregulation of mTOR is involved in AMPK activating autophagy [27]. To confirm whether the mechanism of mangiferin-enhanced autophagy is related to the AMPK signal pathway, the p-AMPK and p-mTOR expression was examined by western blot. Western blot analysis in a time-dependent manner indicated that mangiferin treatment increases p-AMPK expression but does not affect the expression of total AMPK; on the contrary, mangiferin decreases p-mTOR expression (Figures 5(a) and 5(b)). In addition, western blot analysis showed that p-AMPK expression is significantly increased and p-mTOR level is remarkably decreased after treatment with mangiferin in a dose-dependent manner (Figures 5(c) and 5(d)). To determine whether mangiferin-enhanced autophagy is mediated by the AMPK pathway, compound C, an AMPK inhibitor, was used for inhibiting AMPK expression in chondrocyte ([Supplementary-material supplementary-material-1]). As shown in Figures 5(e) and 5(f), mangiferin increased the level of ATG5 and LC3II/I ratio and decreased the level of p62, but administration of compound C abrogated the effect of mangiferin on autophagy. These data demonstrated that the AMPK pathway is involved in mangiferin-activated autophagy.

### 3.6. Inhibition of Autophagy Attenuates Mangiferin-Induced Antiapoptosis and ECM Synthesis

**Figure 6:**
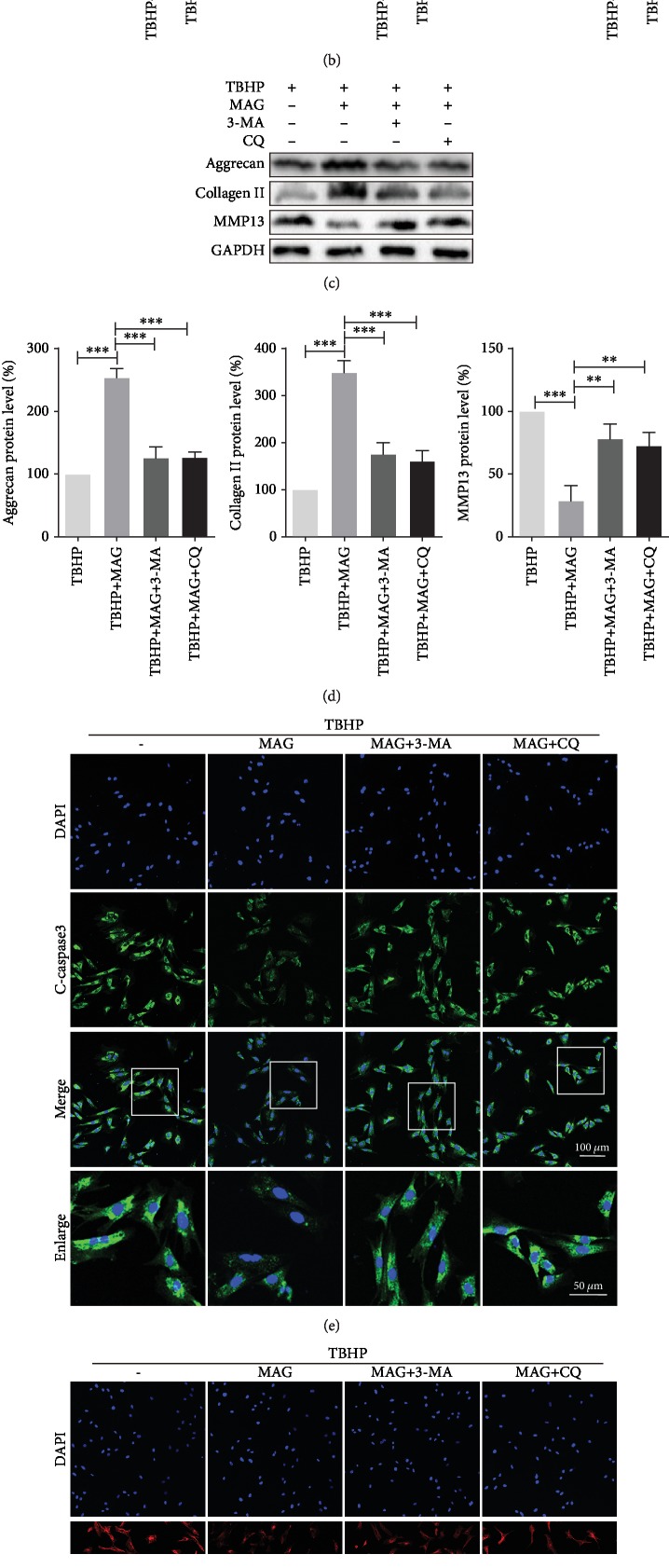


The activation of autophagy is believed to be related to antiapoptosis and ECM synthesis. To confirm further the effects of mangiferin-induced autophagy in protecting chondrocyte survival and ECM synthesis, two autophagy inhibitors, 3-MA and CQ, were used to block autophagy ([Supplementary-material supplementary-material-1]). As shown in Figures 6(a) and 6(b), 3-MA or CQ treatment significantly increases the level of C-caspase3 and Bax and decreases the expression of Bcl-2 in chondrocytes, respectively. On the other hand, western blot analysis showed that the administration of 3-MA or CQ remarkably reduces aggrecan and collagen II expression and enhances MMP13 expression in comparison to the mangiferin treatment group, which indicates that the inhibition of autophagy promotes ECM degradation (Figures 6(c) and 6(d)). In line with western blot analysis, the result of immunofluorescence staining indicated that chondrocytes treated with 3-MA or CQ upregulate C-caspase3 and MMP13 expression compared with mangiferin treatment (Figures 6(e) and 6(f)). Meanwhile, compound C treatment abrogates the antiapoptosis and ECM synthesis effects on chondrocytes ([Supplementary-material supplementary-material-1]). Combining these results, mangiferin attenuating apoptosis and enhancing ECM synthesis is required for the autophagy augmenting.

### 3.7. Mangiferin Ameliorates OA Development in DMM Mouse Model

**Figure 7:**
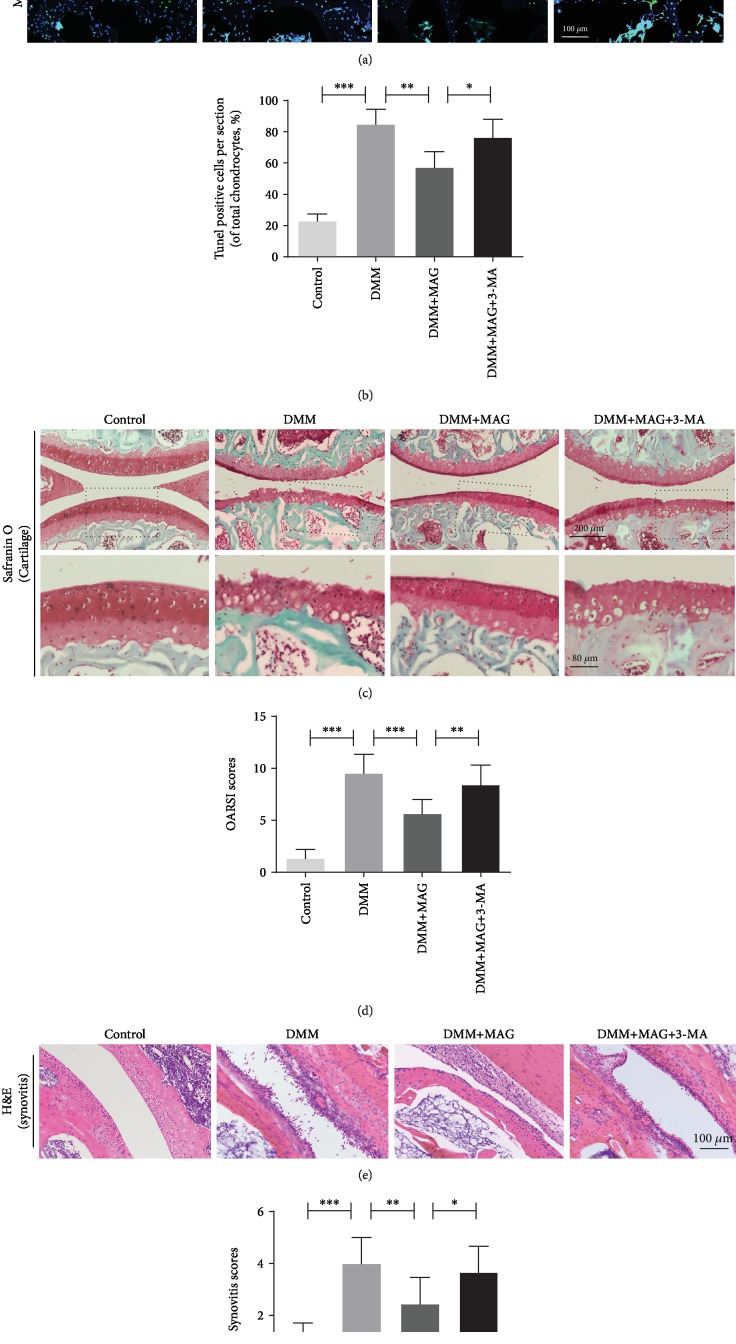


To investigate the potential therapeutic effects of mangiferin on OA in vivo, the mouse was fed with mangiferin and TUNEL staining and Safranin O staining were performed to assess histomorphology differences of mouse knee joints. TUNEL staining revealed the higher proportion of cellular apoptosis in the DMM group than in the control group. Nevertheless, the mangiferin treatment reversed this pathological change (Figures 7(a) and 7(b)). Safranin O staining showed that the OA group presented aberrant narrowing of the joint space after surgery, whereas lower narrowing of joint space was observed in the mangiferin treatment group. Meanwhile, Safranin O staining also indicated the erosion and hypocellularity of the superficial articular cartilage in the OA group. On the contrary, the mangiferin treatment group shows the more complete and smooth cartilage surface and richer proteoglycan when compared with the OA group (Figures 7(c) and 7(d)). Furthermore, the thickening and hypercellularity of the synovium were observed at 8 weeks after surgery by hematoxylin and eosin (H&E) staining, while mangiferin treatment alleviated synovitis compared to the OA group (Figures 7(e) and 7(f)). However, 3-MA treatment reversed the beneficial outcomes induced by mangiferin. Consistent with the above staining, the results of the OARSI and synovitis scores were also increased in the OA group, which were decreased in the mangiferin treatment group.

## 4. Discussion

OA is a complex process involving a variety of pathophysiological mechanisms, including excessive apoptosis of cells and degradation of ECM. And several pharmacological administration or genetic regulations for apoptosis and ECM anabolism show beneficial effects against OA development in the cellular and animal models [4, 6, 28]. However, the potential therapeutic effect of mangiferin on OA has been rarely mentioned. In the current study, we demonstrated that the administration of mangiferin alleviates oxidative stress-induced apoptosis and ECM degradation in mouse chondrocyte and degenerative cartilage. Meanwhile, the result suggested that the molecule mechanism of mangiferin is related to enhancing the level of autophagy and restoring autophagic flux in chondrocyte. Here, we provide insight into the treatment effects and underlying mechanisms of mangiferin and present a potential value for the medical treatment for OA in clinical management.

The pathophysiology of aging-associated degeneration disease is characterized by multiple factor involvement, including inflammatory cytokines, mechanical loading, and metabolic stress, which causes the increased level of reactive oxygen species (ROS) [29]. The elevated ROS is a significant threat for cellular homeostasis and results in the accumulation of damaged DNA and outer membrane destroyed mitochondria, especially in aging with weak antioxidant defense mechanism [30, 31]. The disturbance between the ROS overproduction and antioxidant deficit is highly likely to be a basic contributor to cellular apoptosis and senescence [26, 32, 33]. Currently, we applied TBHP as a ROS donor to irritate the apoptosis and ECM degradation of chondrocyte in an in vitro study. As expected, the administration of TBHP significantly increased the expression of proapoptotic proteins and matrix degradation enzymes and dramatically decreased the viability of chondrocyte, as well as ECM components. Interestingly, mangiferin treatment reverses the above adverse situations induced by TBHP, suggesting that mangiferin plays a protective role for chondrocyte invaded by oxidative stress.

As a key cellular homeostatic mechanism, autophagy is an essentially intracellular catabolic process which responds to increased metabolic demands or stresses, including the increased ROS. As an avascular organ with low rate of cell turnover in the body, the especial environment of articular cartilage forces chondrocytes to adapt to the low oxygen supply by turning to quiescence [13, 34]. Studies showed that chondrocytes adapt to the hypoxic environment dependent on autophagy activation in the normal condition. Moreover, the active autophagy was observed in pathological situations; this response acts as a compensatory protection process for the maintenance of normal cell function and survival through removing mitochondria, peroxisomes, ribosomes, or misfolded proteins [12, 15]. Our results showed that autophagic associated proteins slightly increase following the use of TBHP. On the other hand, the decline in the activity of autophagy was also detected in OA both in human and animal models in a previous study [11]. To this contradiction, we think that long-term aggravating degeneration worsens irreversible autophagy dysfunction with age instead of short-term stimulation in chondrocytes.

Meanwhile, studies reported that the autophagosome formation is reduced owing to the impaired lysosome activity in the process of OA [35]. Consequently, enhancing autophagic flux has been defined as a potential therapeutic strategy for OA. In the present study, the level of p62 was rising after TBHP treatment, suggesting that the autophagic flux was blocked. To solve this issue, we applied mangiferin that is proven to be an autophagy inducer currently. Mangiferin treatment not only increases autophagosome protein formation but also restores lysosome activity and promotes the fusion of autophagosome and lysosome. And the protective effects of mangiferin were abrogated when autophagic flux was inhibited, indicating that mangiferin plays the protective role by enhancing autophagic flux.

A variety of studies focused on the signaling pathways that lead to autophagy under ROS conditions. AMP-activated protein kinase (AMPK) as a metabolic sensor plays a critical role in regulating autophagy [27]. The mechanism of mangiferin-induced autophagy was further explored. In line with these studies, we found that mangiferin-induced autophagy in chondrocytes was related to the phosphorylation of AMPK. As is known, the mTOR pathway is a negative regulator of autophagy, and mTOR activity is involved in AMPK-mediated autophagy. Previous study reported that mTOR regulate chondrocyte differentiation through the parathyroid hormone-related peptide signal pathway, and the inactivation of mTOR is necessary for chondrocytes in the grow plate [20]. Our results showed that mangiferin treatment decreases the mTOR activity both in a time- and dose-dependent manner. Certainly, we note that employed siRNA or knockdown gene instead of molecular inhibitors would be more persuasive. Though this is a limitation, the effect of mangiferin on autophagy is confirmed; it is available to clarify the properties and underlying mechanisms in future researches.

**Figure 8:**
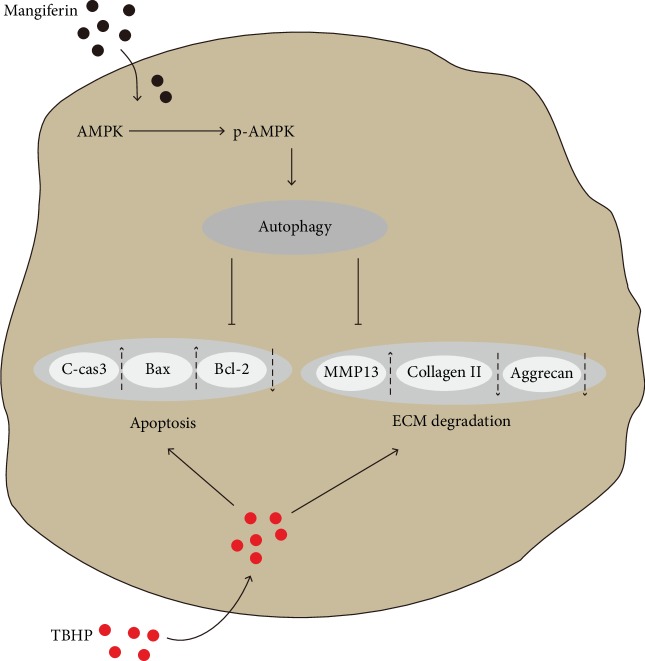


In conclusion, we demonstrated that apoptosis and ECM degradation were upregulated in TBHP-treated mouse chondrocytes. Mangiferin administration targeted activated autophagy and restored oxidative stress-induced autophagic flux disruption in mouse chondrocytes, which was contributed to protect chondrocytes against apoptosis and ECM degradation stimulated by TBHP (Figure 8). In addition, in vivo experiment confirmed the therapeutic effect of mangiferin on OA in the mice model. These findings provide insight into the potential of mangiferin in the treatment of osteoarthritis.

## Data Availability

The datasets used and analyzed during the current study are available from the corresponding authors on reasonable request.

## Supplementary Materials

Supplementary Materials Figure S1: compound C treatment decreases AMPK expression in chondrocytes. (A-B) The protein expression of AMPK in mouse chondrocytes, GAPDH was used as a loading control. All data represent mean values ± SD (*n* = 5); significant differences in different groups are indicated as ^∗^*P* < 0.05, ^∗∗^*P* < 0.01, and ^∗∗∗^*P* < 0.001. Figure S2: 3-MA and CQ treatment inhibits autophagy in chondrocytes. (A-B) The protein expression of LC3 and p62 in mouse chondrocytes, GAPDH was used as a loading control. All data represent mean values ± SD (*n* = 5); significant differences in different groups are indicated as ^∗^*P* < 0.05, ^∗∗^*P* < 0.01, and ^∗∗∗^*P* < 0.001. Figure S3: compound C treatment increases apoptosis and ECM degradation. (A-B) The protein expression of AMPK, Bax, Bcl-2, C-caspase3, aggrecan, collagen II, and MMP13 in mouse chondrocytes; GAPDH was used as a loading control. All data represent mean values ± SD (*n* = 5); significant differences in different groups are indicated as ^∗^*P* < 0.05, ^∗∗^*P* < 0.01, and ^∗∗∗^*P* < 0.001.
