# Supplementary material for: Mangiferin Prevents TBHP-Induced Apoptosis and ECM Degradation in Mouse Osteoarthritic Chondrocytes via Restoring Autophagy and Ameliorates Murine Osteoarthritis
Source: Oxid Med Cell Longev. 2019 Oct 15;2019:8783197. doi: 10.1155/2019/8783197 (PMC6815628; doi:10.1155/2019/8783197)

**Supplementary Figure Legends**

**Figure S1.Compound C treatment decreases AMPK expression in chondrocytes.** (A-B) The protein expression of AMPK in mouse chondrocytes treated with TBHP (25 μM), mangiferin (100 μM) and Compound C (5 μM) for 24h in each group as above, GAPDH was used as a loading control. All data represent mean values ± SD, n=5, significant differences in different groups are indicated as **P*<0.05, ***P*<0.01, ****P*<0.001.

**Figure S2. 3-MA and CQ treatment inhibits autophagyin chondrocytes.** (A-B) The protein expression of LC3 and p62 in mouse chondrocytes treated with TBHP (25 μM), mangiferin (100 μM), 3-MA (2.5 mM) and CQ (25 μM) for 24h in each group as above, GAPDH was used as a loading control. All data represent mean values ± SD, n=5, significant differences in different groups are indicated as **P*<0.05, ***P*<0.01, ****P*<0.001.

**Figure S3. Compound C treatment increases apoptosis and ECM degradation.** (A-B) The protein expression of AMPK, Bax, Bcl-2, C-caspase3, aggrecan, collagen II and MMP13 in mouse chondrocytes treated with TBHP (25 μM), mangiferin (100 μM) and Compound C (5 μM) for 24h in each group as above, GAPDH was used as a loading control. All data represent mean values ± SD, n=5, significant differences in different groups are indicated as **P*<0.05, ***P*<0.01, ****P*<0.001.

**Figure S1**


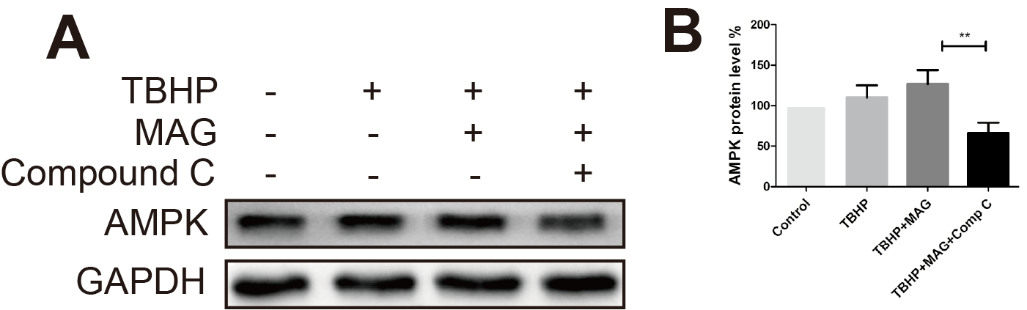


**Figure S2**


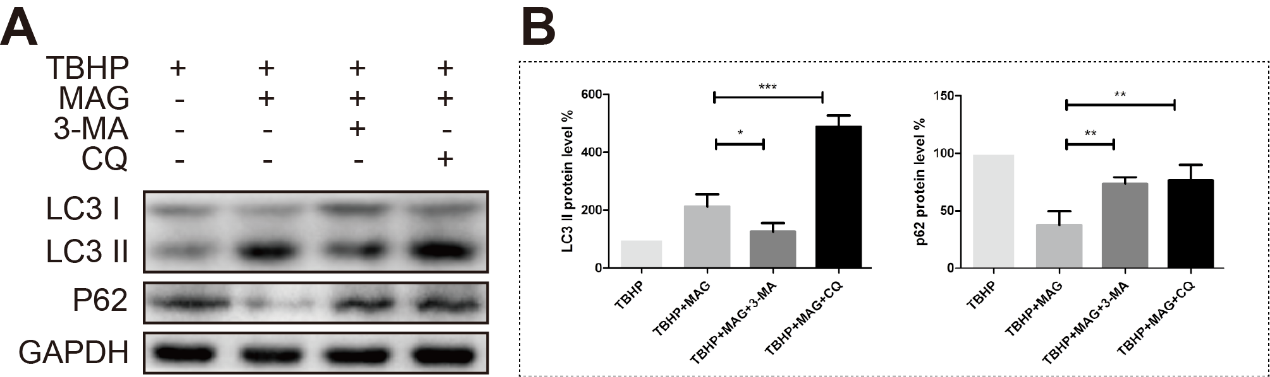


**Figure S3**


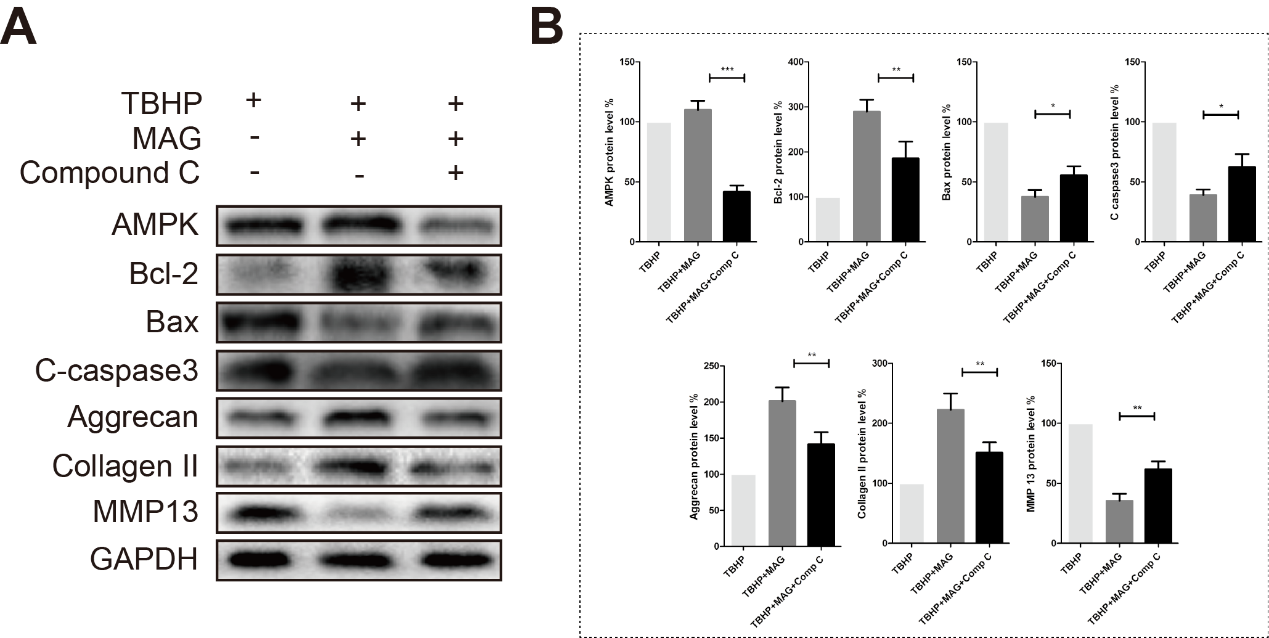

Supplement: Supplementary Materials — Figure S1: compound C treatment decreases AMPK expression in chondrocytes. (A-B) The protein expression of AMPK in mouse chondrocytes, GAPDH was used as a loading control. All data represent mean values ± SD (n = 5); significant differences in different groups are indicated as ∗P < 0.05, ∗∗P < 0.01, and ∗∗∗P < 0.001. Figure S2: 3-MA and CQ treatment inhibits autophagy in chondrocytes. (A-B) The protein expression of LC3 and p62 in mouse chondrocytes, GAPDH was used as a loading control. All data represent mean values ± SD (n = 5); significant differences in different groups are indicated as ∗P < 0.05, ∗∗P < 0.01, and ∗∗∗P < 0.001. Figure S3: compound C treatment increases apoptosis and ECM degradation. (A-B) The protein expression of AMPK, Bax, Bcl-2, C-caspase3, aggrecan, collagen II, and MMP13 in mouse chondrocytes; GAPDH was used as a loading control. All data represent mean values ± SD (n = 5); significant differences in different groups are indicated as ∗P < 0.05, ∗∗P < 0.01, and ∗∗∗P < 0.001. [file 8783197.f1.docx]
